# Stereotactic Lesioning of the Thalamic Vo Nucleus for the Treatment of Writer's Cramp (Focal Hand Dystonia)

**DOI:** 10.3389/fneur.2018.01008

**Published:** 2018-11-26

**Authors:** Takeshi Shimizu, Tomoyuki Maruo, Shimpei Miura, Haruhiko Kishima, Yukitaka Ushio, Satoshi Goto

**Affiliations:** ^1^Department of Neurosurgery, Parkinson's Disease Research Center, KKR Otemae Hospital, Osaka, Japan; ^2^Department of Neurosurgery, Graduate School of Medicine, Osaka University, Osaka, Japan; ^3^Department of Neurodegenerative Disorders Research, Graduate School of Medical Sciences, Institute of Biomedical Sciences, Tokushima University, Tokushima, Japan

**Keywords:** focal hand dystonia (FHD), thalamotomy, thalamus anatomy, movement disorder, writer's cramp

## Abstract

Writer's cramp (focal hand dystonia) is a sporadic focal dystonia that affects a specific part of the upper limb causing excessive co-contraction of antagonistic muscles. It usually presents as a task-specific dystonia, including, among others, writing of a character or playing a musical instrument. Although treatments for writer's cramp exist, medical therapy often results in unsatisfactory outcomes in patients with this type of dystonia. However, accumulating evidence suggests that long-term and complete remission of various types of focal hand dystonia can be achieved with stereotactic ablation or deep brain stimulation of the thalamic ventral-oralis complex (Vo) nucleus, which includes both the ventralis oralis posterior and anterior nuclei of the thalamus. Following the striking therapeutic success of Vo thalamotomy in patients with medically-refractory writer's cramp, we here introduce the use of stereotactic lesioning of the thalamic Vo nucleus for the treatment of this focal type of dystonia. Our findings identified patients with disabling writer's cramp (i.e., it prevents their success in their professional careers) to be good candidates for positive outcome with this surgical technique.

## Introduction

Among dystonia syndromes, focal hand dystonia (FHD) is as a sporadic focal dystonia with adult onset, typically between the ages of 30 and 50 years (1). Although being relatively common, it is less frequent compared to other focal dystonias, including cervical dystonia and blephalospasm (2). FHD affects a specific part of the hand and arm causing an excessive co-contraction of antagonistic muscles and an over-activation of inappropriate muscles (3). Considering that FHD usually manifests as a task-specific dystonia, it can be classified as an occupational dystonia, referred to the writer's, typist's, guitarist's, or pianist's cramp (2, 4). However, as this dystonia also can have a widespread effect on muscles, it can involve different tasks, and, in some instances, it can also progress to the other hand (5).

**Figure 1:**
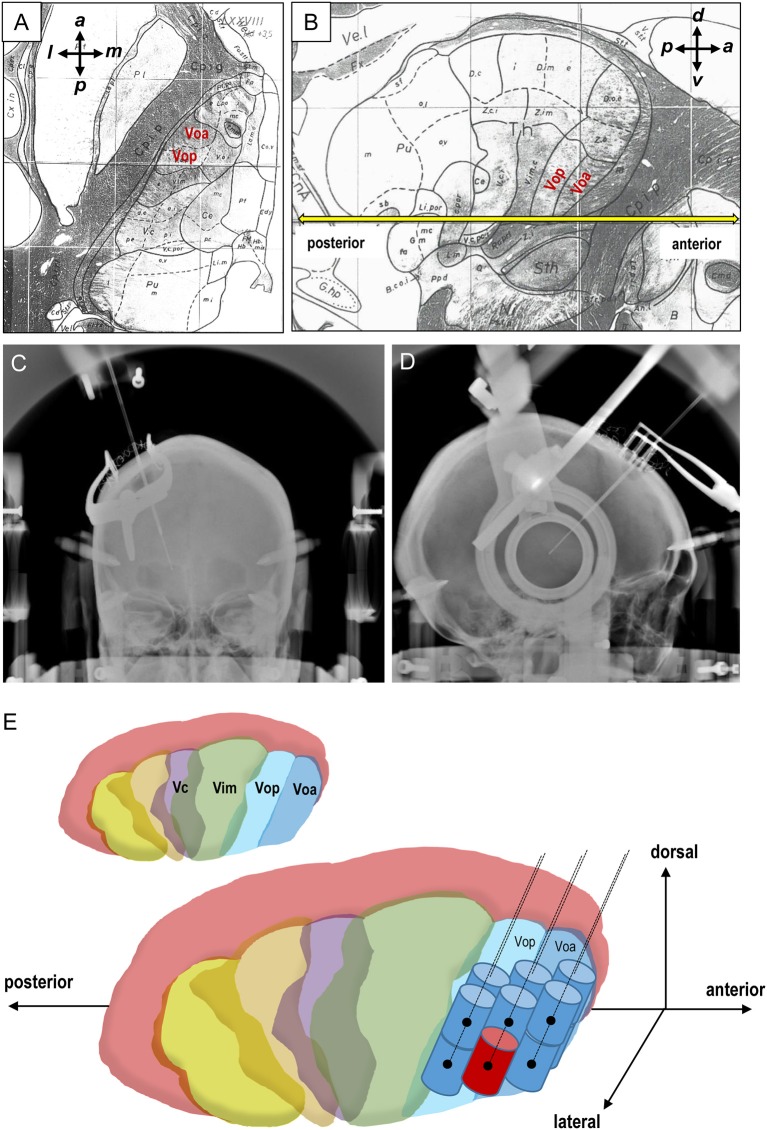
Stereotactic target and lesioning of the thalamic Vo nucleus. **(A,B)** Axial **(A)** and sagittal **(B)** planes from the Schaltenbrand-Wahren atlas. The anterior commissure-posterior commissure (AC-PC) line is colored yellow **(B)**. *a*, anterior; *l*, lateral; *p*, posterior; *m*, medial; *d*, dorsal; *v*, ventral. **(C,D)** Frontal **(C)** and lateral **(D)** views of the radiologic location of the radiofrequency coagulative electrodes implanted in the thalamic Vo nucleus. **(E)** Schematic representation for creation of a total of 12 radiofrequency lesions in the thalamic Vo nucleus. The primary target is marked by red color. Vim, the ventralis intermedius nucleus; Vop, the ventralis oralis posterior nucleus; Voa, the ventralis oralis anterior nucleus.

Although the pathophysiology of FHD is yet to be fully understood, evidence indicates that somatosensory brain circuits dysfunction affects motor coordination in patients with dystonias (6–8). The thalamic ventral-oralis complex (Vo) nucleus, which includes both the ventralis oralis posterior (Vop) and anterior (Voa) nuclei of the thalamus (for reference see Figures 1A,B), plays a key role in the cortex-basal ganglia-thalamus-cortex loop, related to the “motor” function (9). Notably, the pallidothalamic and cerebellothalamic afferents are greatly intertwined within the Vo nucleus, with the Voa being predominantly associated with the pallidothalamic pathway, and the Vop with the cerebellothalamic pathway instead (10). More than two decades ago, we first applied selective Vo thalamotomy as a treatment option for dystonic writer's cramp (11). Ever since, multiple reports have further shown that stereotactic ablation (12–18) or deep brain stimulation (DBS) (19–21) of the Vo nucleus is a successful therapy for patients with various types of FHD. By evaluating a single center case series, we here introduced the use of stereotactic lesioning of the thalamic Vo nucleus for the treatment of writer's cramp.

## Patients and methods

### Patients

Four patients (one man and three women; aged 25 to 44 years) with focal dystonia of the right upper limb underwent Vo thalamotomy at the KKR Otemae hospital in Osaka, Japan (Table 1). All the patients reported symptoms for longer than 2 years, and these were refractory to ordinary medical treatments. None of the patients took prescription medication. According to Sheehy and Marsden's classification (5), whilst two patients suffered from dystonic writer's cramp, the other two presented simple writer's cramp. Preoperative magnetic resonance (MR) images revealed an absence of abnormal findings in all patients. After receiving a detailed explanation of the surgery, all patients provided written informed consent.

**Table 1:** Clinical summary of the patients with writer's cramp who underwent selective Vo thalamotomy.

**Patient**	**Diagnosis**	**Symptom duration**	**Follow-up period**	**Writing movement score (WMS)**		
				**Preoperative**	**7 days after surgery**	**1 year after surgery**
1	Dystonic writer's cramp	2 years	2 years	20	0	0
2	Dystonic writer's cramp	20 years	1 year	22	0	0
3	Simple writer's cramp	4 years	1 year	22	0	0
4	Simple writer's cramp	2 years	1 year	26	0	0

Patients with unilateral Vo thalamotomy were included in this study if they met the following criteria: (1) diagnosis of writer's cramp according to the consensus statement of Albanese et al. (1), (2) men and women, aged at 18–80 years, (3) medically-refractory writer's cramp that prevents their success in their professional careers, and (4) written informed consent to participate in this assessment.

### Clinical assessments

The Writing Movement Score (WMS), which represents a sub-score of the Writer's Cramp Rating Scale (WCRS) (22), was employed for an objective clinical assessment of patients' writer's cramp during the baseline, perioperative, and follow-up visits at 3 month intervals after surgery (Table 1). The writing test was also performed prior to and 7 days following surgery and follow-up visits. A video recorded the manifestation of patients' symptoms during the baseline and follow-up visits. MR images were acquired on the fourth post-operative day and in the subsequent follow-up visits. The surface EMG examination to determine the responsible muscles was routinely performed before the surgery.

### Surgical procedures

All surgical procedures were performed under local anesthesia using Leksell's stereotactic system (Elekta K. K., Stockholm, Sweden) and a surgical navigation system (Medtronic, Minneapolis, MN, USA). The thalamic Vo nucleus was targeted on contrast-enhanced MR images, with the primary target being the Voa-Vop nuclei junction. Its stereotactic coordinates were determined following the Schaltenbrand-Wahren atlas (23) (Figures 1A,B) and reported as 13.5 mm lateral, 2 mm posterior, and 1 mm dorsal to the midpoint of the anterior commissure-posterior commissure (AC-PC) line (11, 20). Additionally, the needle trajectory was also predetermined to avoid the damage of the visible vessels and lateral ventricle. Throughout the operation, x-ray images were taken to provide for the anterior-posterior and lateral views to confirm the accurate placement of the electrode tips onto the target (Figures 1C,D). A monopolar radiofrequency probe (1.0 mm diameter and 2.0 mm uninsulated tip length) was used for electrostimulation testing at 160 Hz, 100 μs, and 3.0 mA, which is fundamental to achieve improvements of symptoms without any adverse effects. Finally, after testing for a lesion at 45°C for 60 s, a permanent anatomic lesion was made by heating the electrode tip to 70°C for up to 60 s. The electrode was moved in increments of 2-mm each, and the lesioning process was repeated to increase the overall lesion size. A total of 12 radiofrequency lesions through 6 tracks were created with the primary target as the starting point, as shown in Figure 1E.

## Results and discussion

**Figure 2:**
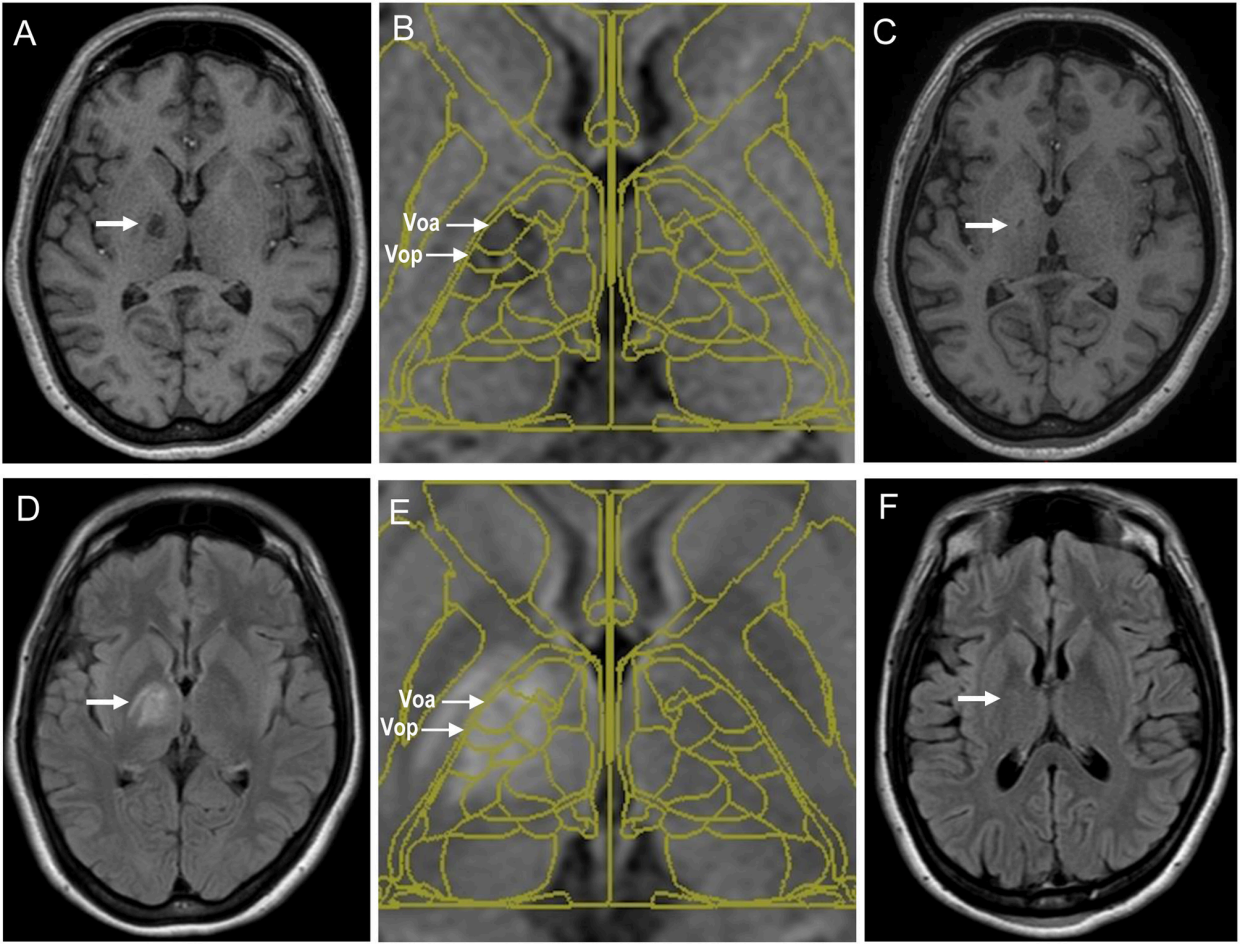
Magnetic resonance images of coagulative lesions located in the thalamic Vo nucleus of a patient with writer's cramp who underwent stereotaxy. **(A–C)** The T1-weighted acquisition images at 4 days **(A)** and 1 year **(C)** after the surgery are shown. Fusion of the Schaltenbrand and Wahren atlas with the T1-weighted acquisition image at 4 days after the surgery is also shown in **(B)**. Coagulative lesions are indicated by arrows **(D–F)**. The fluid-attenuated inversion recovery (FLAIR) images at 4 days **(D)** and 1 year **(F)** after the surgery are shown. Fusion of the Schaltenbrand and Wahren atlas with the FLAIR image at 4 days after the surgery is also shown in **(E)**. Coagulative lesions are indicated by arrows. Voa, the ventralis oralis anterior nucleus; Vop, the ventralis oralis posterior nucleus; Vim, the ventralis intermedius nucleus; Vc, the ventralis caudalis nucleus.

The symptoms of dystonia present in all four patients with writer's cramp completely disappeared following the selective lesioning of the thalamic Vo nucleus. The mean preoperative WMS was 23.3 (range: 22–26). As determined by neurological examinations, which included the WMS and writing tests, patients' symptoms were markedly alleviated within 7 days following surgery. Their writing speed improved markedly and eventually the WMS results reached zero in all patients (Table 1). An absence of recurrence of symptoms was reported throughout the follow-up period (1–2 years). The T1-weighted MR images confirmed that the coagulative lesions were accurately placed in the thalamic Vo nucleus (Figures 2A–C). Notably, the MR images with fluid-attenuated inversion recovery (FLAIR) showed a vasogenic edema surrounding the lesions on the fourth post-operative day (Figures 2D,E), which however almost disappeared at 1–2 years after surgery (Figure 2F). Although none of the patients experienced surgical morbidity or mortality, they all complained of a transient (4 days −1 month) and slight paresthesia of the lower limbs contralateral to the lesions, which however did not lead to walking difficulties. Additionally, an absence of postoperative speech disturbance was reported. Therefore, selective Vo thalamotomy offers a means to achieve a long-term and complete suppression of writer's cramp without causing any serious adverse effect.

Partial symptom relapse after selective Vo thalamotomy is possible, although infrequent, despite the initial improvement (12, 16, 17). This may indicate that sufficient lesioning of both the Vo nuclei, which are around 6–7 mm in size, is necessary to achieve a successful outcome. The coagulative lesions created in this current study, which covered both the Vop and Voa, appeared to be larger in size as compared to those in previous reports (12–18), yet, none of the patients suffered from any permanent complication. Considering that care should be taken to avoid direct injury to the internal capsule or other surrounding structures, intraoperative tests conducted with stimulating electrodes are necessary prior to the lesioning, as described above. All the patients complained of a slight numbness of the contralateral lower extremity, which occurred due to the dysfunction of their posterior limb of the internal capsule. We suggest this adverse event to be caused by the vasogenic edema surrounding the lesions, given that this subjective symptom disappeared within 1-month post-operation, in concordance with the disappearance of the vasogenic edema.

## Concluding remarks

Writer's cramp represents a disabling neurological condition that often interferes with people's ability to succeed in their professional careers. However, the optimal therapy for this focal type of dystonia remains to be established. Considering that a variety of therapeutic interventions, including pharmacotherapy (2), botulinum toxin therapy (24–26), and transcranial magnetic stimulation (7, 27), which have been used to treat writer's cramp, have often achieved unsatisfactory results, the further exploration of surgical interventions for sustained and definite suppression of writer's cramp may be beneficial. This study provides evidence that stereotactic ablation of the thalamic Vo nucleus represents a successful therapeutic strategy, devoid of any permanent complication in patients with writer's cramp. In contrast to a previous report (16), we do not recommend the use of bilateral Vo thalamotomy for the treatment of bilateral writer's cramp. In fact, bilateral thalamotomy implies the potential risk of serious and *irreversible* complications after bilateral lesioning, as suggested for dystonia patients (28–31). We recommend instead the use of bilateral Vo DBS or unilateral Vo DBS together with unilateral Vo thalamotomy for treating bilateral FHD. This notion is supported by the fact that the therapeutic efficacy of Vo DBS for suppressing FHD is almost compatible to that of Vo thalamotomy (19–21). Although greater experience with our surgical technique is needed to confirm the present results, stereotactic lesioning of the thalamic Vo nucleus may represent a surgical option for the treatment of medically-refractory and disabling writer's cramp. To achieve successful outcome, we suggest that appropriate sizing and accurate positioning of the stereotactic lesion may be of utmost importance for both obtaining the greatest benefits and avoiding any surgical complication.

## Ethics statement

This study was carried out in accordance with the recommendations of KKR OSAKA Hospital Institutional Review Board with written informed consent from all subjects. The protocol was approved by the Institutional Review Board.

## Author contributions

TS and SG contributed to the conception and design of the study; writing and revising the manuscript. TS, TM, SM, HK, YU, and SG contributed to data collection and analysis.

### Conflict of interest statement

The authors declare that the research was conducted in the absence of any commercial or financial relationships that could be construed as a potential conflict of interest.

## References

[1] Albanese A Bhatia K Bressman SB Delong MR Fahn S Fung VSC. Phenomenology and classification of dystonia: a consensus update. Mov Disord. (2013) 28:863–73. 10.1002/mds.25475 23649720 PMC3729880

[2] Lin PT Shamim EA Hallett M. Focal hand dystonia. Pract Neurol. (2006) 6:278–87. 10.1136/jnnp.2006.10124622174867

[3] Lin PT Hallett M. The pathophysiology of focal hand dystonia. J Hand Ther. (2009) 22:109–13. 10.1016/j.jht.2008.10.008 19216051 PMC2699180

[4] Stahl CM Frucht SJ. Focal task specific dystonia: a review and update. J Neurol. (2017) 264:1536–41. 10.1007/s00415-016-8373-z 28039522 PMC5502053

[5] Sheehy MP Marsden CD. Writers' cramp-a focal dystonia. Brain (1982) 105(Pt 3):461–80. 10.1093/brain/105.3.461 7104663

[6] Quartarone A Hallett M. Emerging concepts in the physiological basis of dystonia. Mov Disord. (2013) 28:958–67. 10.1002/mds.25532 23893452 PMC4159671

[7] Cho HJ Hallett M. Non-invasive brain stimulation for treatment of focal hand dystonia: update and future direction. J Mov Disord. (2016) 9:55–62. 10.14802/jmd.16014 27240806 PMC4886207

[8] Jinnah HA Neychev V Hess EJ. The anatomical basis for dystonia: the motor network model. Tremor Other Hyperkinet Mov. (2017) 7:506. 10.7916/D8V69X3S 29123945 PMC5673689

[9] Alexander GE Crutcher MD. Functional architecture of basal ganglia circuits: neural substrates of parallel processing. Trends Neurosci. (1990) 13:266–71. 10.1016/0166-2236(90)90107-L 1695401

[10] Morigaki R Nagahiro S Kaji R Goto S Current use of thalamic surgeries for treating movement disorders. In: Justin LS editor. Thalamus: Anatomy, Functions and Disorders. Hauppauge, NY: Nova Scientific Publishers (2011). p. 1–31.

[11] Goto S Tsuiki H Soyama N Okamura A Yamada K Yoshikawa M. Stereotactic selective Vo-complex thalamotomy in a patient with dystonic writer's cramp. Neurology (1997) 49:1173–4. 10.1212/WNL.49.4.1173 9339715

[12] Taira T Hori T. Stereotactic ventrooralis thalamotomy for task-specific focal hand dystonia (writer's cramp). Stereotact Funct Neurosurg. (2003) 80:88–91. 10.1159/000075165 14745214

[13] Shibata T Hirashima Y Ikeda H Asahi T Hayashi N Endo S. Stereotactic Voa-Vop complex thalamotomy for writer's cramp. Eur Neurol. (2005) 53:38–9. 10.1159/000084262 15746551

[14] Horisawa S Taira T Goto S Ochiai T Nakajima T. Long-term improvement of musician's dystonia after stereotactic ventro-oral thalamotomy. Ann Neurol. (2013) 74:648–54. 10.1002/ana.23877 23463596

[15] Asahi T Koh M Kashiwazaki D Kuroda S. Stereotactic neurosurgery for writer's cramp: report of two cases with an overview of the literature. Stereotact Funct Neurosurg. (2014) 92:405–11. 10.1159/000366004 25359570

[16] Horisawa S Goto S Nakajima T Kawamata T Taira T. Bilateral stereotactic thalamotomy for bilateral musician's hand dystonia. World Neurosurg. (2016) 92:585.e21–5. 10.1016/j.wneu.2016.05.017 27188636

[17] Doshi PK Ramdasi RV Karkera B Kadlas DB. Surgical interventions for task-specific dystonia (Writer's dystonia). Ann Indian Acad Neurol. (2017) 20:324–7. 10.4103/aian.AIAN_15_17 28904473 PMC5586136

[18] Asahi T Taira T Ikeda K Yamamoto J Sato S. Full recovery from drummer's dystonia with foot and arm symptoms after stereotactic ventro-oral thalamotomy: a case report. Acta Neurochir. (2018) 160:835–38. 10.1007/s00701-018-3480-5 29423776

[19] Fukaya C Katayama Y Kano T Nagaoka T Kobayashi K Oshima H. Thalamic deep brain stimulation for writer's cramp. J Neurosurg. (2007) 107:977–82. 10.3171/JNS-07/11/0977 17977270

[20] Goto S Shimazu H Matsuzaki K Tamura T Murase N Nagahiro S. Thalamic Vo-complex vs. pallidal deep brain stimulation for focal hand dystonia. Neurology (2008) 70(Pt 2):1500–1. 10.1212/01.wnl.0000310430.00743.11 18413578

[21] Cho CB Park HK Lee KJ Rha HK. Thalamic deep brain stimulation for writer's cramp. J Korean Neurosurg Soc. (2009) 46:52–5. 10.3340/jkns.2009.46.1.52 19707494 PMC2729825

[22] Wissel J Kabus C Wenzel R Klepsch S Schwarz U Nebe A. Botulinum toxin in writer's cramp: objective response evaluation in 31 patients. J Neurol Neurosurg Psychiatry (1996) 61:172–5. 10.1136/jnnp.61.2.172 8708685 PMC1073991

[23] Schaltenbrand G Wahren W Atlas for Stereotaxy of the Human Brain With an Accompanying Guide. Stuttgart: Thieme (1977).

[24] Lungu C Karp BI Alter K Zolbrod R Hallett M Long-term follow-up of botulinum toxin therapy for focal hand dystonia: outcome at 10 years or more. Mov Disord. (2011) 26:750–3. 10.1002/mds.23504 21506157 PMC3081109

[25] Karp BI Das C Truong D Hallett M Treatment of focal hand dystonia. In: Truong D Hallett M Zachary C editors. Manual of Botulinum Toxin Therapy, 2nd Edition. New York, NY: Cambridge University Press (2013). p. 71–84.

[26] Jackman M Delrobaei M Rahimi F Atashzar SF Shahbazi M Patel R. Predicting improvement in writer's cramp symptoms following botulinum neurotoxin injection therapy. Tremor Other Hyperkinet Mov. (2016) 6:410. 10.7916/D82Z15Q5 27625900 PMC5013165

[27] Erro R Tinazzi M Morgante F Bhatia KP. Non-invasive brain stimulation for dystonia: therapeutic implications. Eur J Neurol. (2017) 24:1228–e64. 10.1111/ene.13363 28782903

[28] Andrew J Fowler CJ Harrison MJ Stereotactic thalamotomy in 55 cases of dystonia. Brain (1983) 106:981–1000. 10.1093/brain/106.4.981 6360306

[29] Yamashiro K Tasker RR. Stereotactic thalamotomy for dystonic patients. Stereotact Funct Neurosurg (1993) 60:81–5. 10.1159/000100593 8511436

[30] Cardoso F Jankovic J Grossman RG Hamilton WJ. Outcome after stereotactic thalamotomy for dystonia and hemiballismus. Neurosurgery (1995) 36:501–8. 7753350 10.1227/00006123-199503000-00009

[31] Yoshor D Hamilton WJ Ondo W Jankovic J Grossman RG. Comparison of thalamotomy and pallidotomy for the treatment of dystonia. Neurosurgery (2001) 48:818–24. 11322442 10.1097/00006123-200104000-00025

